# Tribological Effects of Surface Biomimetic Micro–Nano Textures on Metal Cutting Tools: A Review

**DOI:** 10.3390/biomimetics10050283

**Published:** 2025-05-01

**Authors:** Zhenwen Sheng, Hui Zhu, Yu He, Bo Shao, Zhi Sheng, Suqin Wang

**Affiliations:** College of Engineering, Shandong Xiehe University, Jinan 250109, China; shengzhenwen@sdxiehe.edu.cn (Z.S.); zhuhui@sdxiehe.edu.cn (H.Z.); heyu@sdxiehe.edu.cn (Y.H.); shaobo@sdxiehe.edu.cn (B.S.)

**Keywords:** surface microtextures, biomimetics, tribological properties, cutting tools

## Abstract

Surface microtexture, as a branch of surface engineering, has always been an active research object due to its ability to significantly improve matrix properties. Especially by combining surface microtextures with biomimetics, the concept of surface microtextures has been greatly expanded. The emergence of biomimetic microtextures has also endowed mechanical components with better tribological properties and longer service life. This article reviews the preparation techniques of surface microtextures and summarizes the advantages and limitations of various microtexture preparation techniques. We discuss the morphologies of different biomimetic microtextures and the unique properties they impart to the substrate surface, explore the influence of biomimetic microtexture morphology and size parameters on their tribological properties, and reveal the mechanism of biomimetic microtextures applied to cutting tool surfaces. Finally, the application of biomimetic microtextures in cutting tools is prospected.

## 1. Introduction

Frictional wear phenomena are ubiquitous in life, especially in engineering practice, and the reduction in energy consumption due to friction has long been a research direction [1]. However, the use of lubricants poses several problems, such as environmental pollution, so researchers have been building typical microscopic profiles at friction interfaces to achieve friction reduction and wear reduction [2,3]. Surface texturing is a possible solution for improving the tribological properties of mechanical components [4]. Over the last few decades, significant progress has been made in surface texturing technology, which is seen as a viable option for surface engineering, significantly improving the load-bearing capacity and wear resistance of tribomechanical components and reducing the coefficient of friction [5], thus contributing to the sustainable manufacturing of parts and the functionalization of surfaces.

Over the years, various surface texturing techniques have been developed that can add or remove the corresponding material from the surface of a part [6], such as abrasive processing [7], reactive ion etching [8], electron beam texturing [9], electrical discharge texturing [10], etc. Each technique has its advantages and disadvantages in terms of flexibility, accuracy, cost of texturing, and speed of processing. Several researchers have theoretically and experimentally demonstrated that surface texturing can reduce friction and wear in a variety of applications such as pistons/cylinders [11,12,13,14,15,16,17,18,19,20], mechanical seals [21,22,23,24,25,26], cutting tools [27,28,29,30,31,32,33,34,35,36,37,38,39,40], hydraulic motors [41,42,43], bearings [44,45,46,47,48,49], cams/tappets [50], and prosthetic joints [51,52,53,54]. Several surface texture forms are mainly mentioned in the literature: micro-depressions [55,56,57], micro-stripes [58], micro-grooves [59], and banded grooves [60]. In addition, researchers have prepared raised surface textures, which are effective in reducing the contact area of the friction interface and thus reducing the adhesion to achieve a lower coefficient of friction [61].

Extensive research confirms that surface texture tribological performance exhibits strong dependence on morphological design and parametric optimization [62,63,64,65,66,67,68,69,70,71,72,73,74]. Advanced manufacturing technologies now enable the precise fabrication of sophisticated textured architectures. Geometric configuration exerts a critical influence on friction interface functionality, governed by pattern dimensional characteristics. Morphological diversity in texture design expands the solution space for tribological challenges [75].

**Figure 1:**
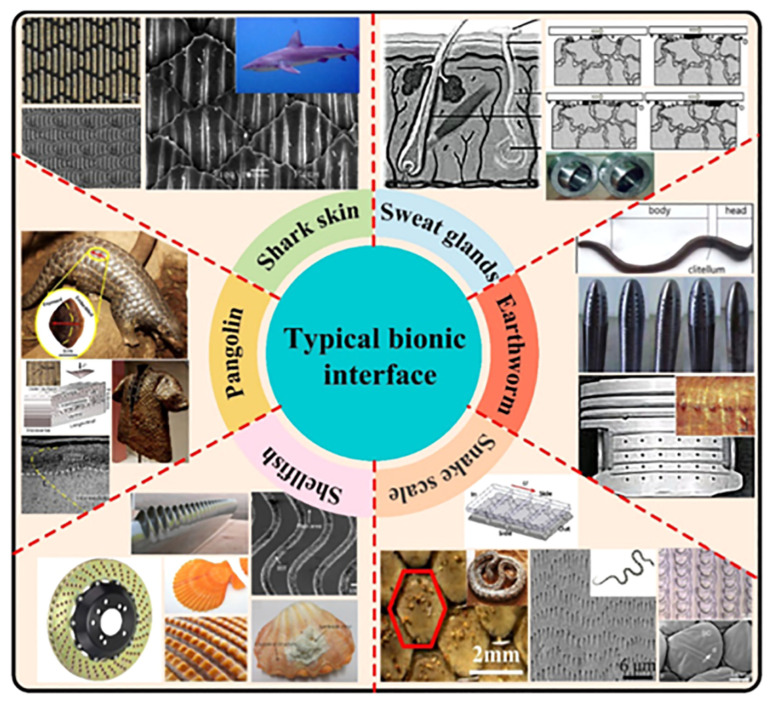
Typical biological structures and biomimetic applications. Reprinted by Ref. [85], 2022, Elsevier.

Bio-inspired engineering disciplines systematically transfer biological system principles to technological innovation [76]. Evolutionary processes spanning hundreds of millions of years have yielded tribologically optimized architectures in zoological specimens (Figure 1), including serpentine integuments, annelid cuticles, mollusk exoskeletons, and elasmobranch dermal denticles. These natural prototypes demonstrate exceptional extreme-environment adaptability, serving as paradigms for reverse-engineering biological superlative properties through multidisciplinary analytical methodologies [77,78]. The excellent tribological properties exhibited by these biological structures have led to a new field of research—biomimetic tribology—where biological mechanisms of friction reduction, wear, and efficient lubrication are investigated to produce biomimetic structures and materials with similar effects [79]. The influence of various biomimetic structures on improving the tribological properties of materials has been studied theoretically and experimentally by many researchers at this stage [71,80,81,82,83,84]. Therefore, sorting out and analyzing various biological structures in nature with low friction and wear resistance properties and their mechanisms and summarizing the current situation of the application of various biomimetic materials in the field of tribology will better promote the development of biomimetic tribology.

While previous studies have extensively explored surface texturing techniques and their tribological effects, most reviews remain confined to traditional microstructures (e.g., dimples and grooves), lacking a critical analysis of the unique advantages offered by biomimetic microtextures. Furthermore, the existing literature fails to systematically address the selection of texture manufacturing methods for large-scale production. Additionally, research on microtextured cutting tools predominantly focuses on traditional microtextures, with limited discussions on the impact of biomimetic microtextures in this context. This paper comprehensively summarizes the preparation methods, advantages, and limitations of biomimetic micro–nano textures while identifying approaches suitable for industrial applications. It subsequently details naturally occurring biomimetic surface textures with superior tribological properties and their lubrication mechanisms, explores the influence of texture morphology and parameters on tribological performance, and concludes by reviewing current applications of biomimetic textures on metal cutting tools while proposing future design directions.

## 2. Common Preparation Methods for Biomimetic Micro–Nano Textures

The excellent tribological properties of biomimetic textures have attracted a great deal of attention both in academia and in industry. The preparation of biomimetic textures involves chemical and physical methods for the biomimetic processing of concave and convex structures with a specific shape and array distribution on the surface of the material. The following is a brief description of the new methods used to create biomimetic textures in recent years and a summary of their advantages and disadvantages.

### 2.1. Laser Processing Technology

**Figure 2:**
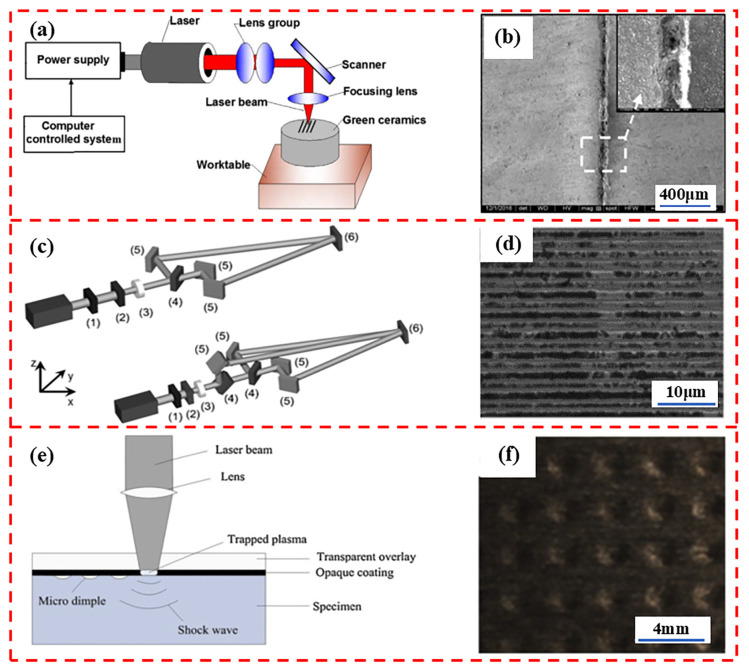
Schematic diagrams and corresponding micrographs of laser ablation processing (**a**,**b**), laser interference processing (**c**,**d**) and laser impact processing (**e**,**f**). Reprinted by Ref. [90], 2017, Elsevier. Reprinted by Ref. [91], 2015, Wiley. Reprinted by Ref. [92], 2014, Elsevier.

Compared to other subtractive processing techniques, laser processing has attracted considerable interest in recent years due to its superior flexibility, selectivity, accuracy, efficiency, and ability to produce customized surfaces with different wettability, adhesion, and friction properties [86]. Typically, laser processing techniques are dominated by laser ablation, but in recent years, new laser-based material processing processes, such as laser interference [87] and laser impact processing [88] (Figure 2), have emerged as alternative methods for expanding the impact of laser surface treatment techniques for tribological applications [89].

Li et al. [27] employed laser surface restructuring to fabricate lattice-patterned microchannels with varied spacing dimensions, achieving a 16% friction coefficient reduction and a 70% wear rate decrease relative to non-textured specimens. Xing et al. [84] engineered Si_3_N_4_/TiC ceramic surfaces with biomimetic grooves, replicating crocodilian dermal scutes, crustacean exoskeletal features, elasmobranch placoid scales, teleost fish opercular structures, and lepidotrichia patterns. Liu et al. [90] implemented 1064 nm pulsed laser ablation protocols for generating microscale groove architectures on unfired ZrO_2_ ceramic substrates. Aguilar et al. [91] demonstrated the laser-interferometric fabrication of high-aspect-ratio linear/columnar periodic microstructures on austenitic stainless steel surfaces. Bieda et al. [92] developed 1D/2D periodic micropattern arrays on 100Cr bearing steel through direct laser interference lithography. Choi et al. [93] established laser interference patterning capabilities for creating uniform nanodot arrays on monocrystalline silicon wafers. The production of textural features at a high resolution is the most significant advantage of laser interference techniques. Li et al. [94] fabricated an array of micro-pits on copper by laser impact machining techniques. It was found that the surfaces treated by the laser impact machining technique exhibited better tribological properties as well as reduced wear and adhesive wear compared to the untreated surfaces. Yakimets et al. [95] showed that the use of laser impact machining techniques could reduce the wear rate of rolled 100Cr steel by 33%. Lim et al. [96] demonstrated that laser shock peening elevated the microhardness of duplex stainless steel from 250 HV to 310 HV, correlating with a 16% reduction in wear rate. While laser texturing is widely adopted for surface modification due to its rapid processing, flexibility, and controllability [71], thermal effects inherent to both direct laser ablation and interference methods induce adverse consequences, including material degradation, phase transformations, and tensile residual stresses, which detrimentally alter surface topography and mechanical properties [84]. Although laser shock processing enhances wear resistance through surface hardening, its serialized fabrication microscopic features on a case-by-case basis results in inefficiency, limiting industrial scalability [97]. Additionally, laser-based techniques are incompatible with materials exhibiting atypical optical responses [71].

To improve the accuracy of laser processing technology, femtosecond lasers are the next advanced method after picosecond and nanosecond lasers for laser processing technology [98]. Femtosecond-pulsed laser systems employ broadband spectral synchronization to generate ultrashort optical pulses. The extended bandwidth (>10 nm) enables sub-100 fs pulse durations, attaining peak power levels exceeding 15 GW [99]. When compared to nanosecond or picosecond laser processing, femtosecond systems exhibit substantially reduced thermal impact regions and minimal particulate deposition at ablation sites, thereby achieving enhanced machining precision [100].

### 2.2. Reactive Ion Etching

**Figure 3:**
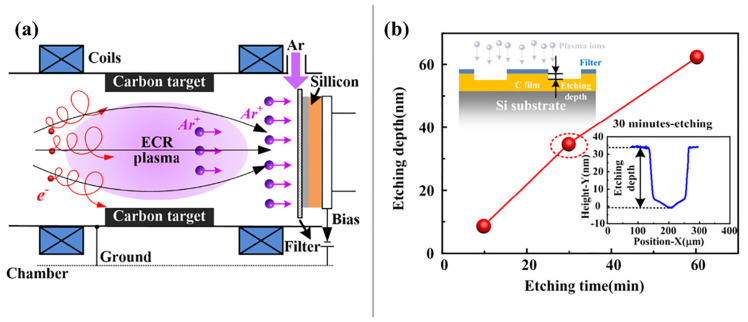
Schematic illustrations of the etching process (**a**) and the relationship between etching time and etching depth (**b**). Reprinted by Ref. [103], 2019, Elsevier.

Reactive ion etching (RIE) combines physical ion bombardment and chemical reactions for surface patterning [101]. In this process, inert gas ions are accelerated in a low-vacuum environment to form a focused beam that interacts with the workpiece surface, while reactive species generated in the chamber induce chemical etching, achieving material removal through dual mechanisms [8]. To fabricate textures via RIE, a patterned mask is applied to the substrate prior to ion irradiation, enabling localized etching [102]. Chen et al. [103] developed a filter-assisted ion etching method to create crater textures on carbon films (Figure 3), demonstrating optimal tribological performance at a 30 nm depth and 10 μm diameter. This configuration reduced the friction coefficient by 73.9% and increased wear life by 40 times compared to untextured carbon films. Similarly, Wang et al. [23] applied RIE to generate micro-pit arrays on SiC surfaces, significantly enhancing water-lubrication efficiency and expanding the low-friction operational range.

Reactive ion etching has the advantages of a fast etching speed and high etching quality. In laboratory experiments, the RIE technique is a suitable choice for the preparation of small-sized and high-precision microtextures. In research in recent years, the RIE technique has mostly been applied to crystal surfaces such as single-crystal silicon. However, there are some problems in the processing of the RIE technique, such as the presence of more obvious damage to the material surface, the lack of precision in the control of the ion beam, the harsh experimental environment, and expensive experimental equipment [71]. In addition, the overall production cycle of the technology is long, and the process is complex. Therefore, the RIE technology at this stage is still not suitable for industrial use in terms of cost and efficiency.

### 2.3. Soft Lithography

Soft lithography is a technique that uses elastic impressions, molds, and conformal photomasks to fabricate or replicate structures. It is called soft because it uses elastic materials, most notably polydimethylsiloxane (PDMS) [104,105]. Mahmoud et al. [106] prepared UV-curable electroactive polyurethane acrylate materials with a superhydrophobic surface structure, mimicking a peacock feather using PDMS as a template, and applied it to anti-corrosion coatings. Wang et al. [107] prepared nickel films with positive and negative textured surfaces with lotus and rice leaf patterns. The prepared nickel films were superhydrophobic and had excellent tribological properties after chemical treatment. Ryu et al. [108] successfully replicated a new microstructured PDMS coating with high durability and relatively low friction from a lotus leaf using soft lithography. The results showed that the frictional wear of the microstructured PDMS specimens was significantly lower than that of the smooth specimens and that the extremely high durability was attributed to the dissipation of frictional energy through the elastic deformation of the microstructure. The benefits of soft lithography include not only a relatively low cost, easier setup, and higher efficiency, but also pattern resolution from nanometer to micron accuracy. A disadvantage of soft lithography is the need to use other methods such as photolithography or electron beam lithography to create stamp masters. Since it is difficult to prepare masters for patterns on animal body surfaces, most research at this stage on soft lithography for the preparation of biomimetic textures has focused on the preparation of biomimetic patterns in the form of plant surfaces (e.g., lotus flowers [109], rice leaves [107], etc.).

**Figure 4:**
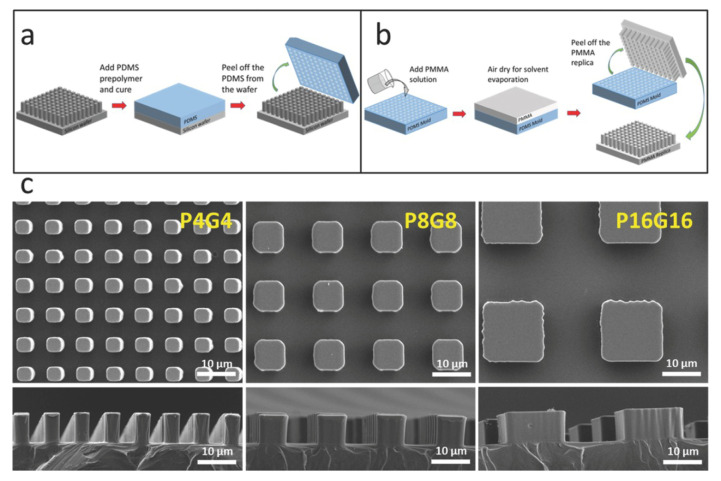
Common soft lithography microfabrication technology processes. (**a**) Fabrication of the PDMS molds of the silicon master templates. (**b**) Construction of the PMMA replicas of the master templates on the PDMS molds by solvent-casting method. (**c**) Top (**upper row**) and side (**lower row**) view SEM images of the micropillar-decorated PMMA films. Reprinted by Ref. [113], 2016, Wiley.

In addition to the basic soft lithography methods, several additional patterning methods based on embossing, molding, and embossing of elastomeric stamps using soft lithography have been developed in recent years, including phase shift edge lithography [110], nano-transfer printing [111], and polymer pen lithography [112]. Figure 4 shows common soft lithography microfabrication techniques.

### 2.4. 3D Printing

**Figure 5:**
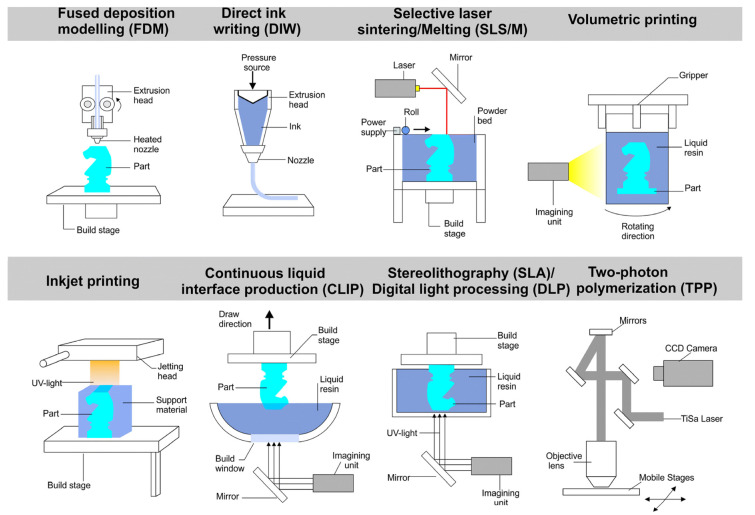
Schematic representation of 3D printing technology. Reprinted by Ref. [121], 2020, Royal Society of Chemistry.

**Figure 6:**
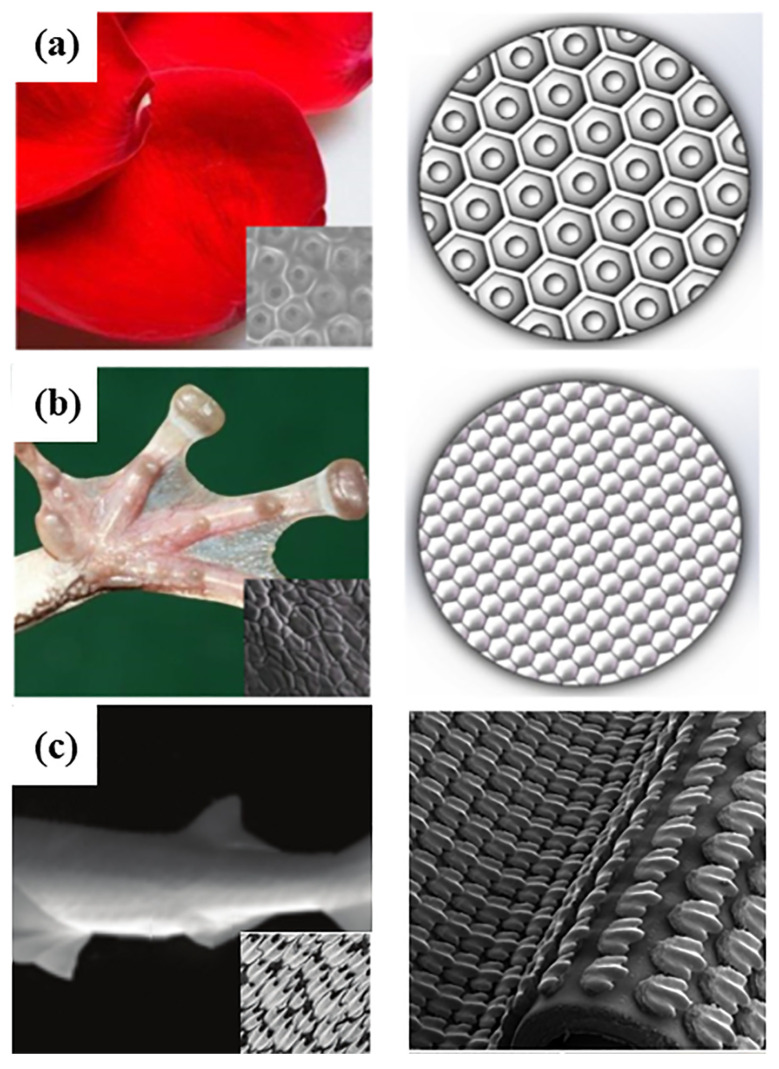
Different biomimetic microtextures prepared by 3D printing technology. Flower petals (**a**), tree frog (**b**), and shark skin structures (**c**). Reprinted by Ref. [117], 2020, Elsevier. Reprinted by Ref. [119], 2014, Company of Biologists Ltd.

3D printing is one of the additive manufacturing technologies, a digital model-based technique in which the expected materials are stacked or combined by a CNC system to produce the structure of interest [114] (Figure 5). Hong et al. [115] used a reciprocating friction tester to evaluate the friction and wear properties of different surface textures produced by 3D printing and showed that surface textures can be applied to 3D-printed parts to improve their frictional wear properties. Banik et al. [116] used 3D printing to simulate, design, and fabricate the morphology of frog toe pads and found that a biomimetic-layered mosaic hexagonal model could provide a design solution for future tire treads with enhanced wet friction properties. Chen et al. [117] used an Al_2_O_3_ ceramic slurry to 3D print a frog with a petal and tree structure of biomimetic-textured ceramics, as shown in Figure 6a,b. The surface texture can store lubricants and debris to improve the lubrication properties of the material. Zhao et al. [118] prepared ceramics with a serpentine scale structure using a 3D printer based on an alumina ceramic slurry, and the prepared Al_2_O_3_ ceramics had strong mechanical properties. Wen et al. [119] investigated the hydrodynamic properties of a 3D-printed shark skin (Figure 6c), and the study results showed that certain movement procedures showed higher swimming speeds and lower energy consumption. 3D printing methods are also used to construct parts, patterns, and molds with fine detail for a wide range of applications, and 3D-printed texture manufacturing offers the advantages of being faster, more flexible, and cheaper than traditional techniques [120].

Currently, printing materials are mainly plastics, resins, plaster, ceramics, sand, and metals, and there are very limited materials that can be used for 3D printing. Although many homogeneous and heterogeneous materials have been developed for use in 3D printing, the need to develop new materials still exists, and several new materials are being developed. This need encompasses two dimensions: firstly, not only is there a need for an in-depth study of material–process–structure–property relationships that have already been applied to clarify their advantages and limitations; secondly, there is a need to develop new testing processes and methods to extend the range of available materials. In addition, if the surface of the object to be manufactured is rounded, this can result in deviations in accuracy.

### 2.5. Discussion

Several of the surface texturing techniques discussed above that have been commonly used in recent years are still mainly based on subtractive manufacturing, with the most widely used being laser processing techniques. Most researchers have used LST to create textures on tools as it is environmentally friendly, dimensionally accurate, and offers a high degree of control over shape and size. However, laser processing techniques can create heat-affected zones, resulting in defects in the prepared surface textures. When techniques such as femtosecond lasers are used, the effects caused by the heat-affected zone are greatly reduced, which provides the opportunity to produce microstructures with greater dimensional accuracy and a significant reduction in surface defects. However, the price/performance ratio in the actual industrial production is the nonnegligible factor. Although femtosecond lasers can provide a smaller heat-affected zone and higher precision, the expensive equipment negates its benefits in actual production. Therefore, the nanosecond laser system has become the first choice in industrial implementation, which can not only fabricate the functional surface but also maintain the best price/performance ratio. Moreover, the nanosecond laser system has a faster process compared with femtosecond lasers, which can further improve the production efficiency. In industrial production, if only one or several types of textured surfaces are produced on a large scale, soft lithography is a feasible solution. By investing a significant amount of funds to purchase the stamp master in the early stage, the obtained stamp master can be used multiple times in subsequent production. Overall, the lower material cost and higher production efficiency of soft lithography give it the potential for industrial promotion. In contrast, owing to the immature processes, reactive ion etching and 3D printing techniques are mostly used to prepare textured surfaces in the laboratory. Therefore, from the perspective of the price/performance ratio, these two technologies are not yet suitable for industrial production.

Each of these techniques has its advantages and limitations, as described in Table 1. When the application scenario for which the texture is to be prepared is clear, the manufacturing technique needs to be chosen wisely based on the advantages and disadvantages of each technique to meet the accuracy requirements of the texture and to consider the cost of preparation. When selecting a texture preparation technique, the main considerations should be accuracy and repeatability; no damage to the substrate; high productivity; acceptable preparation costs; compatibility with the geometry and dimensions of the workpiece being processed; and compatibility with the size and shape of the desired texture [122].

The economic characteristics of surface texturing, including pre-treatment costs, equipment costs, and the energy requirements of the process, also need to be considered before it can be widely used in industrial production. However, not only do different methods of surface texture preparation lead to changes in economic characteristics, but multiple parameters within the same method can also lead to increased costs, such as the mask required for the preparation process, processing time, substrate material, inspection, texture size, workpiece shape, and the required accuracy [122]. In addition, there are many factors that cannot be specifically quantified, such as changes in technical development requirements, rapid advances in texturing technology, changes in geography, etc., and it is not possible at this stage to accurately estimate the costs required for any texturing method.

## 3. Common Biological Surface Textures and Their Mechanisms

After hundreds of millions of years of evolution, there are many organisms in nature with a variety of surface textures. A deeper look at these textures reveals that they provide plants and animals with good mechanical and tribological properties, making them better able to survive in nature. After decades of research, the surface textures of organisms with good tribological properties can be broadly classified into six categories: snakes and other reptiles, sharks and aquatic organisms, dung beetles and insect-like organisms, tree frogs and geckos, shellfish, and plants.

### 3.1. Snakes and Other Reptiles

**Figure 7:**
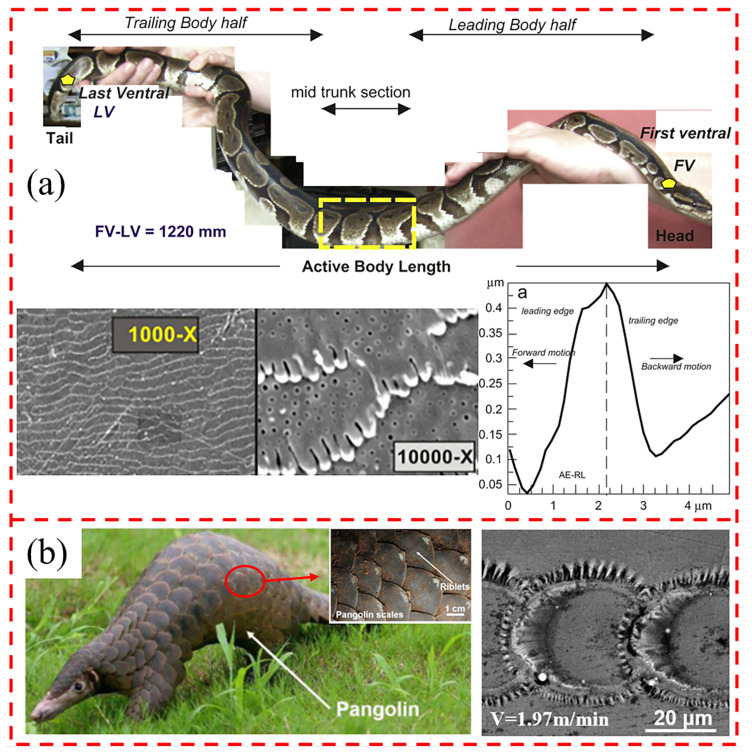
(**a**) Profile of the snake, SEM images of the surface scales, and the outline of the fiber tips along the different measurement axes; (**b**) SEM images of the outer surface of the scales of the pangolin and the corresponding microstructure. Reprinted by Ref. [126], 2011, IOP Publishing. Reprinted by Ref. [130], 2007, Elsevier.

The peculiar physiology of snakes has attracted the attention of researchers due to their lack of limbs and their ability to slide forward at a relatively fast speed. It has been found that the ventral side of the snake’s body is in almost continuous contact with the substrate during locomotion, so their skin may be suitable for generating propulsive forces (high friction) while sliding along the substrate with fairly low friction [124]. This suggests that the ventral scales of snakes have anisotropic frictional properties, with the coefficient of friction depending on the direction of sliding: values during forward movement are lower than those during reverse movement [125]. The frictional behavior has its roots in the structure of the snake’s surface, and the fibers in the scales help the animal to regulate its frictional response. The fibers are asymmetrical, and the slope of the fibril tip is progressively higher from head to tail. This asymmetrical, tip shape provides directional resistance to movement, with less resistance to snake movement in the forward linear direction compared to the reverse [126] (e.g., Figure 7a). This provides new ideas for the design of biomimetic surface structures that can be used to control friction by controlling changes in the kurtosis and asymmetry of the surface structure.

The desert lizard, also a reptile, has evolved a multilayered skin with diamond-shaped and centrally convex scales tightly covered over softer connective tissue that can effectively reduce wear and tear. This hard/soft composite has both rigid and flexible structural features, creating a coupling between structure, morphology, and material that provides high wear and corrosion resistance [127]. Hoskins et al. [128] prepared a microtextured surface of a desert lizard and found that this texture could control adhesion during friction, while its abrasion width was reduced under all loads. In addition, the biomimetic lizard texture synergized with the lubricant to form a dense and homogeneous lubrication layer at the interface, providing excellent friction and wear reduction under specific operating conditions [129]. The corrugations on the scales of the pangolin, which normally live alone in soil caves, are often worn by soil and rocks and have a corrugated surface (e.g., Figure 7b), a property that has been investigated by researchers to reduce wear under free abrasive wear conditions [130]. Sun et al. [131] simulated the biological properties of the cuticle of pangolin scales and prepared biomimetic units on the surface of graphite cast iron with different unit materials using a laser fusion process. The results showed that the TiC-coated biomimetic specimens had the best wear resistance and showed less wear in the biomimetic unit area than in the untreated area. Li et al. [132] prepared electrosurgical blades with biomimetic pangolin scales, resulting in hydrophobicity and a lower coefficient of friction, and the anti-adhesive properties of the pangolin scale texture could effectively reduce soft tissue adhesion during surgery. Zhang et al. [133] improved the wettability of individual TiO_2_ film surfaces by simulating the perforated scale texture on the TiO_2_ texture surface by means of electrohydrodynamic atomization, thereby improving the adhesion strength of MoS_2_ materials in alternating TiO_2_-MoS_2_ soft and hard films.

### 3.2. Sharks and Aquatic Organisms

Aquatic organisms have evolved different skin textures to achieve improved performance or reduced drag. As the top predator in the ocean, sharks improve swimming speed due to their scales having a directional groove structure that keeps swirls away from the skin surface and allows water to flow easily through the skin, greatly reducing drag in the water [134]. In addition to the drag reduction effect, biomimetic shark skin textures can also be used as an option to improve the tribological properties of material surfaces. Lu et al. [135] demonstrated a shark skin-textured surface made by laser processing and verified through pin-disk friction experiments and theoretical analysis methods that this textured surface can effectively improve the load-bearing capacity of the lubricating film and hence the frictional properties. When shark skin-textured surfaces are used in industry, they have the function of reducing the resistance to stored abrasive chips and secondary lubrication. Li et al. [136] achieved reduced friction coefficients and reduced adhesion/abrasive wear when applying a biomimetic shark skin structure to a ZrO_2_/WS_2_ composite coating. The uniformly distributed diamond-shaped grooves not only reduce the frictional area, but the micro-grooves embedded in the diamond-shaped grooves also collect abrasive chips.

**Figure 8:**
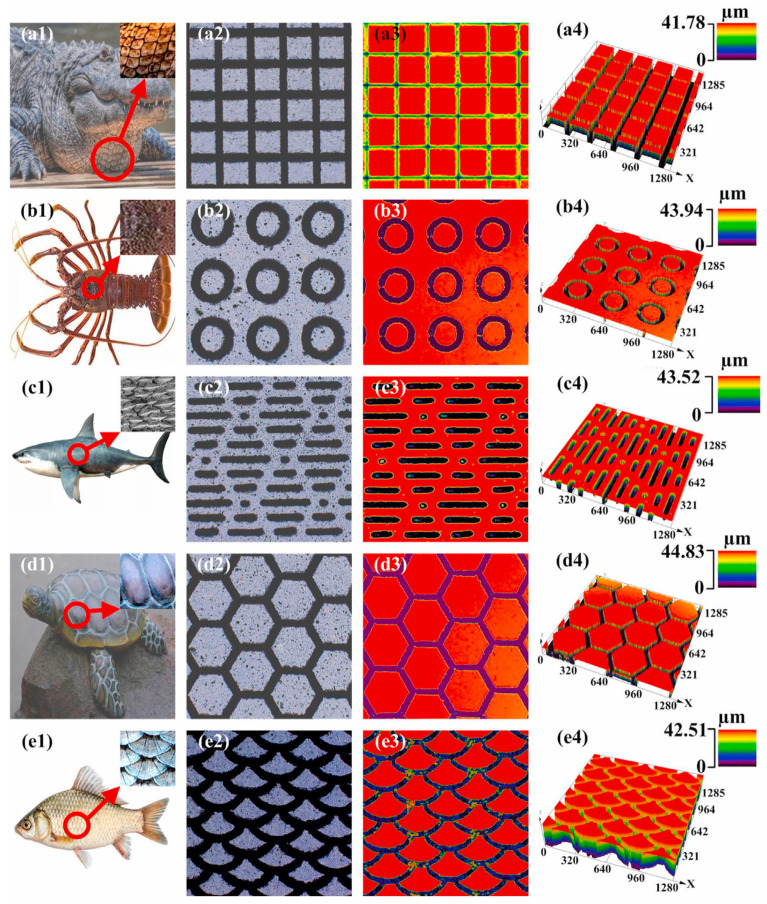
2D and 3D morphology of biomimetic microtextures. (**a1**–**a4**) Crocodile skin square texture; (**b1**–**b4**) lobster shell round texture; (**c1**–**c4**) shark skin diamond texture; (**d1**–**d4**) turtle shell hexagonal texture; and (**e1**–**e4**) fish scale scalloped texture. Reprinted by Ref. [84], 2021, Elsevier.

The surface of the fish is very diverse. In many bony fish, the scales may have a complex surface texture, for example, with protuberances, ridges, and comb-like extensions [137]. Wang et al. [138] prepared a pattern of biomimetic carp scale morphology on Ti6Al4V surfaces, revealing that under different lubrication conditions, the friction coefficient of samples with biomimetic carp scales increased differently as the size of the biomimetic texture increased. In addition, a variety of microtextures can evolve on the surfaces of different aquatic organisms, such as crocodile bellies, lobster shells, shark skins, turtle shells, and fish scales, as well as the corresponding square microtextures, round microtextures, diamond microtextures, hexagonal microtextures, and fan-shaped microtextures were produced [84] (e.g., Figure 8).

The biomimetic textures of aquatic organisms, due to their excellent tribological properties as well as their good hydrodynamic properties, have led to their use in engineering applications involving lubricating media, such as piston cylinders and bearings for enhanced hydrodynamic lubrication, as well as in pipelines for transporting fluids or in housings with fluid dampening properties [139]. The diversity of fish scale types and textures, along with the tribological impact of a rich variety of aquatic surfaces, is a promising area for future research.

### 3.3. Dung Beetles and Insect-like Organisms

The surface microstructure of dung beetle sheath wings is non-smooth and concave, and some researchers have measured their friction coefficients to be between 0.037 and 0.079 using a micro friction meter. They found that the surface texture of the sheath wings and their microstructure dimensions contribute to the anti-friction effect [140]. You et al. [141] prepared a biomimetic tool based on the microstructure of dung beetle heads, and the biomimetic surface significantly reduced the Ti6Al4V dry friction and friction coefficient at the tool–chip interface during cutting. By applying the dung beetle biomimetic texture to the tool surface, it was found that the friction reduction mechanism was that the biomimetic texture could reduce the tool–chip contact areas while improving the ability to capture debris to reduce wear [142].

The head of ants consists of a regular distribution of striped ridges, from which a corresponding striped biomimetic texture can be derived. Su et al. [143,144] used biomimetic laser textures to create striped textures of striped ridges on the head of ants to improve the wear resistance of semi-circular arcs of trailer brake shoes. It was also shown that wear resistance was also related to stripe texture orientation and stripe spacing. Badler et al. [145] investigated the tribological properties of micro-mushroom-shaped biomimetic structures bonded to surfaces observed in combination with potato beetles and spiders. Other researchers have studied grasshoppers, crickets, and other insects that are good jumpers and found that their hind leg femur–tibial joints have a unique surface and texture, with a coefficient of friction of 0.053 ± 0.001 on the coupled surface [146].

### 3.4. Tree Frogs and Geckos

**Figure 9:**
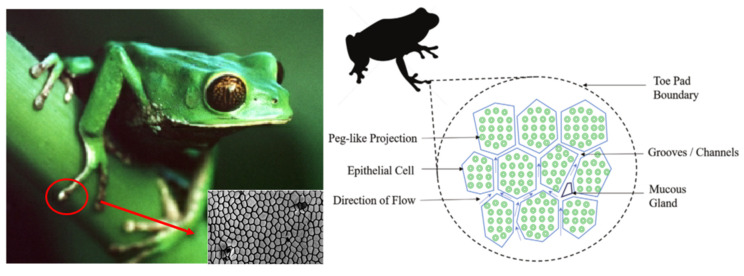
Schematic diagram of the structure of the toe pad of a tree frog. Reprinted by Ref. [116], 2020, Wiley.

Tree frogs are known for their ability to use the pads of their large toes to cling to smooth surfaces. The eighteen toes of tree frogs (four on the forelimbs and five on the hindlimbs) consist of disc-like pads at the tips [147,148]. Their toe pads are heavily lined with epithelial cells, most of which are hexagonal in shape (measuring approximately 10 µm) [149] and separate from each other at the top (e.g., Figure 9). The function of the epithelial cells is to keep the pad firmly attached to an irregular surface. The grooves act like drainage channels and help to distribute the secreted mucus evenly and remove excess water between the pad and the surface to obtain a better contact area to adapt to the irregular surface of the environment [150]. Based on this property, Chen et al. [117] prepared a tree frog toe-end structure by 3D printing and found that this structure had a high coefficient of friction along with good wear resistance. Hexagonal surface patterns are a friction-oriented feature that can inhibit stick-slip and water-slip while achieving friction tuning. Huang et al. [85] showed a significant interaction between the microstructure and the lubricant by using a regular hexagonal microtexture and filling the microtexture with a lubricant. The frictional effect of the biomimetic hexagonal surface texture on the lubricated skin depends on the efficiency of the drainage network that draws the fluid out of the interface and the size of the contact established by the surface projection [151].

Although gecko feet have the same ability to increase friction and adhesion, unlike tree frogs, where adhesion is mainly generated by the micro bristle structure of the foot and van der Waals forces at the interface of the contact surface, the microstructure of the gecko foot sole is mainly uniformly distributed and has a cylindrical structure [152]. Huang et al. [153] found that increasing the area density of the strut pattern also increased the sliding friction. The gecko biomimetic texture is mainly used in robotic hand grasping surfaces [154].

### 3.5. Shellfish

The clam’s prominent shell surface texture prevents chunks of sand from coming into direct contact with the body surface and is resistant to erosion [155]. The spaces between the wave patterns accumulate soft silt and moss, which can reduce wear and tear on the shell surface. In addition, the undulating structure of the wave texture increases the contact area and has a cushioning effect on impact forces, which helps the clam avoid lethal attacks [156]. The functions possessed by these surface textures are important to explore for the optimization of the frictional contact interface, and the spaces between the textures can be filled with the corresponding solid lubricants to play a synergistic role [157]. Lu et al. [158] prepared a composite surface structure of Ni_3_Al-based biomimetic textures and soft and hard solid lubricants concerning the shell surface textures and investigated its tribological properties. The results showed that the biomimetic composite surface structure improved the tribological properties of the material and enhanced the synergistic effect of the surface microtexture and the solid lubricant. Qin et al. [159] prepared the corresponding biomimetic wave texture filled with the SnAgCu-WS2 composite solid lubricant on the surface of TC4, and the results showed that the coupling effect of the biomimetic wave texture and the composite solid lubricant significantly improved the tribological properties. The results showed that the coupling effect between the biomimetic wave texture and the composite solid lubricant significantly improved the frictional wear performance of TC4, and the wave biomimetic texture could improve the solid lubricant deposition efficiency.

### 3.6. Plants

**Figure 10:**
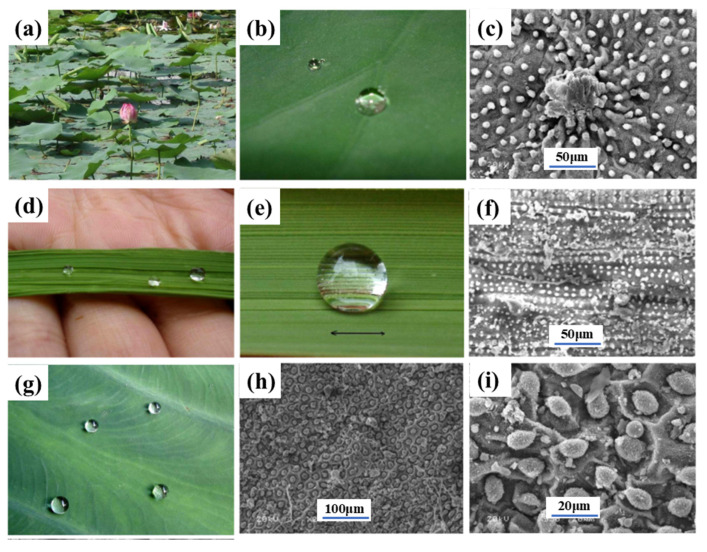
Photographs of some lotus leaves on a pond (**a**) and of water droplets resting on the leaves (**b**) and SEM images of the leaves (**c**); photographs of some water droplets on rice leaves (**d**) and of water droplets resting on rice leaves (**e**) and corresponding SEM images (**f**); photographs of some water droplets on taro leaves (**g**) and SEM images of rice leaves at different magnifications (**h**,**i**). Reprinted by Ref. [169], 2009, Royal Society of Chemistry.

The lotus flower is known for its ability to detach itself from dirty water and expel water from its leaves, making it a symbol of purity in many Asian cultures. The superhydrophobic and self-cleaning mechanism of the lotus leaf is due to the special layered rough profile of its surface in combination with the wax coating (e.g., Figure 10a–c), known as the ‘lotus effect’ [160]. Wang et al. [161] prepared a composite microstructure of diamond-like films with a “lotus leaf” microtexture. The synthesized flexible superhydrophobic diamond-like films with a biomimetic microtexture can be used as an effective lubrication layer, which will be beneficial for many applications. Singh et al. [162] reported on the simulation of lotus and taro leaf surfaces in the case of polymer films. The frictional properties of the replicated surfaces were improved, and the coefficient of friction was reduced by a factor of four compared to the non-replicated surfaces. In addition to lotus leaves as plant biomimetic texturing templates, rice (e.g., Figure 10d–f), taro (e.g., Figure 10g–i), and rose petals (e.g., Figure 6a) can be used as some typical examples of the biological templates being used [163,164,165,166,167,168].

By studying the surface morphology of water-repelling blades, tribologists design and create hydrophobic surfaces to reduce small-scale inter-surface adhesion due to water condensation. The reduction in adhesion can aid easy movement between tiny components in microdevices such as micro and nanoelectromechanical systems. Traditionally, small devices such as MEMS are made of silicon, but the higher interfacial energy (hydrophilicity) of silicon makes it a poor material for friction. Therefore, Yoon et al. [170] produced nanoscale patterns that mimic protrusions on polymer films coated on silicon wafers by soft lithography. These nanopatterned surfaces were hydrophobic (water contact angle of ~99°) compared to bare silicon wafers (water contact angle of ~22°) and had superior tribological properties compared to bare silicon planes. In addition to MEMS, researchers in the biomedical field have used plant biomimetic textures to improve the hydrophobicity of scalpels or scaffolds while avoiding blood contamination [171].

## 4. Effect of Texture Morphology and Parameters on Their Tribological Properties

With a wide variety of biological surfaces covered with micro and nanostructures, nature provides a wide range of models for surface texture design, and different texture shapes and parameters can lead to different tribological properties. Similarly, lubricants exhibit different effects on surfaces with different microtextures.

**Figure 11:**
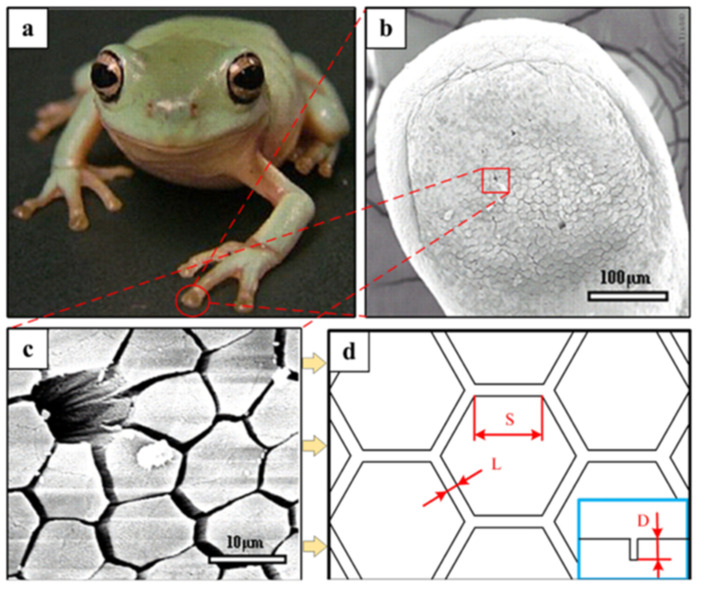
Picture of a juvenile tree frog (**a**), low magnification SEM image of the entire foot pad of a juvenile frog (**b**), high magnification SEM image of the upper skin of the toe pad (**c**), schematic diagram of the optimally designed biomimetic hexagonal texture (**d**). Reprinted by Ref. [172], 2020, Elsevier.

Zhan et al. [157] developed nickel-based MoS_2_ coatings with sinusoidal surface patterns mimicking shell morphology, systematically analyzing the correlation between texture spacing and convexity on interfacial adhesion. Their results showed that appropriate texture spacing and protrusion height significantly improved coating-substrate bond strength, enhancing tribological properties. Qin et al. [156] optimized shell-inspired surface textures on a TC4 alloy using a response surface methodology. Huang et al. [172] designed tree frog-inspired hexagonal textures on AISI 4140 steel (Figure 11), with optimized parameters of 730 μm side length, 360 μm spacing, and 490 μm depth. This biomimetic texture promoted uniform lubricant dispersion and strengthened the lubricant film–substrate bond.

The basic size and spacing of the textures affect their synergy with the solid lubricant more than anything else. The optimum texture size and spacing allow the solid lubricant to form a good lubricating film at the friction interface. However, the texture density is an important parameter that affects its own tribological properties. The texture density is the area occupancy of the textured elements on the friction subsurface [71]. Huang et al. [153] studied a columnar patterned surface with high area density and found that it maintained high friction at high sliding speeds. Zhang et al. [173] found that UHMWPE with a 29.9% high area density texture consistently provided good friction reduction. In addition, the increase in texture density also improved the corrosion resistance of the texture surface [174].

It is clear from the above literature that the influence of the texture parameters on the tribological properties is not independent but is more a result of the interaction with other parameters. No consistent conclusions have been drawn regarding the design of biomimetic texture parameters; therefore, different design choices have to be made for different situations when applying different morphological biomimetic textures. However, researchers can use mathematical or statistical methods (e.g., response surface methodology) to determine the optimal range of parameters that will give the best tribological performance of the surface texture. In contrast to conventional micro–nano texturing, the influence of material matrix parameters on the performance of the texture has been neglected in the current phase of research on biomimetic texturing, and parameters such as surface roughness and material properties, which are easily neglected, can be considered in the design of experiments based on previous research.

The presence of microtextures not only affects the tribological properties of the matrix but also affects the lubrication effect of lubricants. When using liquid lubricants such as lubricating oil or water-based lubricants, the presence of microtextures can cause cavitation effects and promote fluid dynamic pressure lubrication [175]. Moreover, the use of composite/biomimetic microtextures can better increase the average pressure in the microtexture area, reduce the friction coefficient, and achieve stronger fluid dynamics effects [176]. For the process of depositing solid lubricants on textured surfaces, the mechanical interlocking effect of microtextures can effectively improve the bonding strength of solid lubricant coatings, thereby extending the wear life of solid lubricants [28]. Owing to the unique morphological characteristics of biomimetic microtextures, compared with traditional microtextures, more lubricating materials can be preserved during the friction process and achieve secondary lubrication, enabling lubricants to achieve excellent wear life [177]. Some researchers have added solid lubricants as lubricating phases to the cladding layer, and the cladding layer has a microstructure morphology [178]. By ball milling, self-melting powder can be coated on the surface of lubricant nanosheets, thereby preventing solid lubricants from decomposing during the melting process. Even if solid lubricants decompose, they will react with the materials in the cladding layer to generate new phases with lubricating effects [78].

## 5. Biomimetic Textures on Metal Cutting Tools

Metal cutting processes constitute a fundamental pillar of manufacturing engineering, particularly in contemporary mechanical fabrication systems. These operations enable precise component fabrication through controlled material separation, where tool edges mechanically dislodge excess material (chips) from workpieces. The inherent hardness and strength characteristics of metallic alloys subject cutting tools to extreme stress conditions involving both mechanical compression and frictional heat generation. These operational challenges accelerate tool degradation through abrasive wear mechanisms and workpiece material adhesion phenomena, collectively constraining production rates and operational tool durability. To address these problems, introducing surface microtextures into cutting tools to improve the flow and contact of chip materials at the tool–chip interface has become one of the main solutions [179]. Some scholars have introduced biomimetics into the design of tools and designed some biomimetic metal cutting tools for different machining methods to effectively improve their cutting performance.

At this stage, the research on biomimetic texturing in metal cutting tools is divided into two main categories: one is to reduce the contact stress between the tool and the material by changing the microscopic shape of the tool surface only, thus reducing wear or friction; the other is to combine biomimetic texturing with lubricants in a way that uses the synergy between the two to improve tool life or machining efficiency.

**Figure 12:**
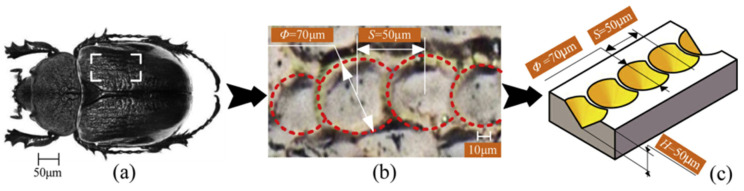
Optical image of the dung beetle (**a**), optical image of the microstructure of the dung beetle’s back (**b**), and topological dimensions of the microstructure of the dung beetle’s back (**c**). Reprinted by Ref. [142], 2021, Elsevier.

**Figure 13:**
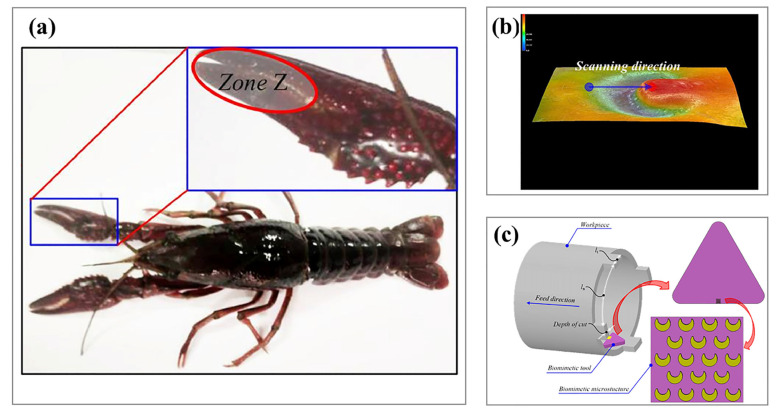
The biomimetic lobster microtexture design originates from the biological reference in panel (**a**), incorporates its three-dimensional morphological characterization in panel (**b**), and demonstrates the structural configuration of intermittent cutting tools employing this texture in panel (**c**). Reprinted by Ref. [182], 2019, SAGE Publications Ltd.

Current turning tool surfaces integrate bio-inspired designs replicating biological pit/groove configurations, demonstrating enhanced wear resistance and machining precision compared to conventional tools [180,181]. While pit/groove patterns remain prevalent, recent advancements explore zoological morphologies including reptile scale arrangements and insect wing venation patterns for optimizing chip evacuation and thermal management during cutting processes. Ni et al. [142] applied dung beetle surface texturing to broaches, where the biomimetic texturing reduced the actual contact area, which in turn reduced friction to lower the coefficient of friction; it also maximized the heat dissipation area of the tool, which then reduced the tool–chip adhesion length (Figure 12). You et al. [141] applied a dung beetle biomimetic microstructure to the front tool face and conducted dry cutting experiments, which showed that the biomimetic surface could significantly improve the cutting performance of the tool. Cui et al. [182] designed a biomimetic laser-induced ceramic tool based on a crayfish epidermal structure, as shown in Figure 13. This biomimetic tool can alter the chip flow during interrupted cutting to reduce the friction between the chip and the tool, thus helping to improve the wear resistance of the tool. Biomimetic microtextures are not only applied to turning tools but can also effectively improve the performance of milling cutters, drill bits, and grinding wheels. Li et al. [183] used a multilevel fuzzy comprehensive evaluation approach to demonstrate that microtextured milling cutters have superior cutting performance compared to traditional milling cutters. Zhang et al. [184] established a finite element model of biomimetic ball-end milling cutters, providing a theoretical basis for the selection of preparation parameters for biomimetic ball-end milling cutters. Lei et al. [185] applied the fitted curve of the rake face of beaver teeth to the rake face of a cylindrical milling cutter, and the newly prepared cylindrical milling cutter showed a better force-saving effect. For the application of biomimetic microtextures on drill, bamboo rat teeth and dung beetle backs have been used as inspirations. The prepared biomimetic microtexture drills have shown better cutting performance and can effectively prevent damage during processing [186,187]. Yu et al. [188] incorporated a fish-scale shape and phyllotaxis arrangement and applied the obtained combined microtextures to a grinding wheel. The research results indicate that the combined biomimetic microtextured grinding wheels effectively guided the flow of the grinding fluid, reduced the waste of grinding fluid, and achieved effective cleaning of the grinding area while ensuring the surface quality of the workpiece.

**Figure 14:**
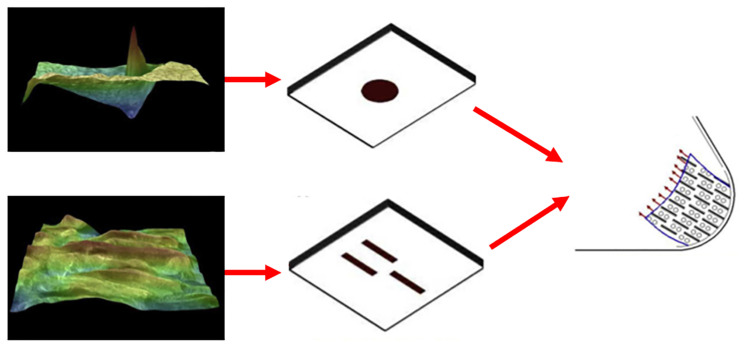
Composite biomimetic-textured surface of dung beetle and bullhead shark. Reprinted by Ref. [189], 2020, Elsevier.

**Figure 15:**
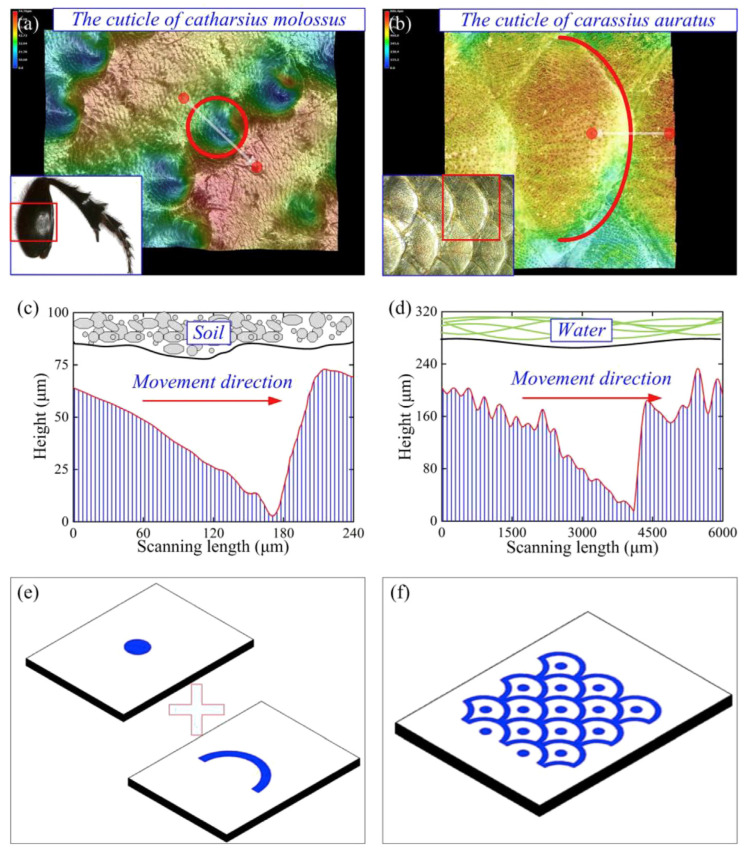
Optical images of scales of dung beetles and carp (**a**,**b**), cross-sectional profiles of two biomimetic structures (**c**,**d**), and topological and combined morphologies of two biomimetic microtextures (**e**,**f**). Reprinted by Ref. [190], 2019, Elsevier.

Based on the multiple biomimetic elements of different organisms, researchers have designed microforms containing multiple biomimetic elements on the same tool surface. Dung beetles and bullhead sharks can control the direction of soil and water flow; based on this feature, Cui et al. [189] developed a ceramic turning tool with a composite biomimetic microscopic surface (e.g., Figure 14) to allow easy chip flow from the workpiece, thereby reducing the thermal load on the tool and improving tool wear resistance. In the same year, Cui also applied the surface micromorphology of a dung beetle and a carp to the surface of a tool (e.g., Figure 15) [190]. The cutting performance of these types of combined biomimetic turning tools has been shown to be superior to that of tools inspired by individual biomimetic elements, for example, by reducing the contact area at the tool–chip interface, thereby achieving lower cutting forces and reducing the thermal load on the tool.

However, not all microtextured turning tools perform better than typical tools during the cutting process, and the influence of microtextures on tool cutting performance under different cutting parameters also needs to be considered. Previous studies have shown that textured cutting tools can exhibit significant effects at low feed rates, and the influence of the microtexture on the cutting debris morphology is limited [191,192]. In addition, different microtexture shapes will have different effects on cutting tools. In general, the pit texture exhibits better performance than the groove texture, especially in harsh environments [193]. When the microtexture size exceeds the effective range, the presence of the microtexture does not improve cutting performance but rather adversely affects it [72]. Xie et al. [194] tested three textured biomimetic turning tools and showed through cutting experiments that the groove microtexture reduces the front tool face wear, with the groove microtexture perpendicular to the chip flow having the best effect, but the fan texture had no significant anti-wear effect. This suggests that the friction reduction effect of textures is not only related to the type of texture but also the direction of the microtexture. Non-smooth biomimetic surfaces are not universally applicable to the design of tools and cutting conditions must be considered when designing biomimetic turning tools.

## 6. Prospects for Biomimetic Texturing on Metal Cutting Tools

**Figure 16:**
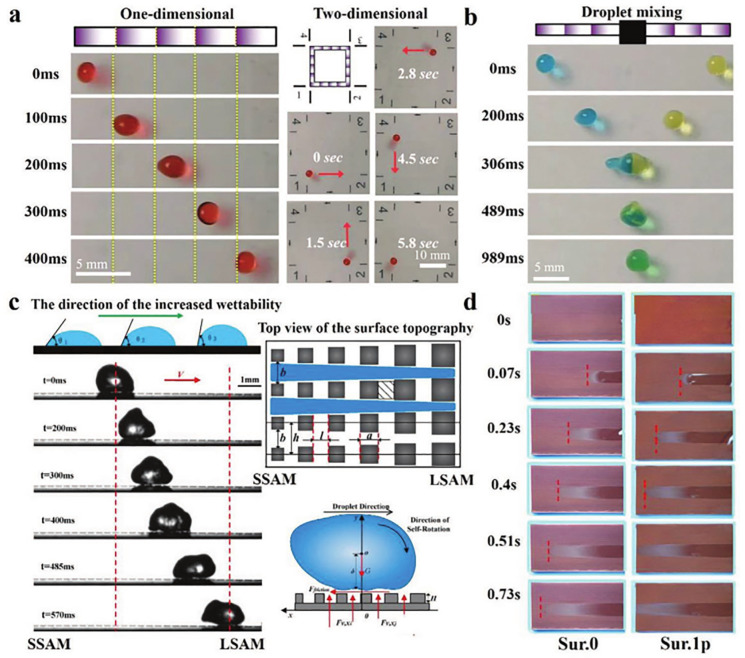
Directional transport of microfluidics on surfaces with different wettability gradients (**a**,**b**), force analysis of droplet movement on textured surfaces (**c**), and droplet movement traces (**d**). Reprinted by Ref. [195], 2016, Trans Tech Publications Inc.

Wetting control is one of the main functions of biomimetic texturing, which can be achieved by constructing different biomimetic texturing on the tool surface to achieve different areas of hydrophilicity and hydrophobicity to provide better lubrication of the cutting fluid during the cutting process and thus improve tool life. In addition, by constructing different scales of texturings on the tool surface, a self-transporting surface can be formed on the tool surface, which allows the cutting fluid to be directed to areas of severe wear. Gradient surfaces have attracted extensive research interest due to their potential for microfluidics, mist/water harvesting, and water electrolysis. At this stage, researchers have been inspired by nature to construct gradient surfaces on the surface of substrates by laser machining to achieve directional microfluidic transport through gradient forces (e.g., Figure 16) [195]. The application of gradient surfaces to tools to achieve directional cutting fluid delivery has important research implications for the use of trace amounts of cutting fluids and for improving tool life.

For dry cutting tools, there have been many literature reports on the influence of microtextures on the tribological or cutting properties of substrates without lubricants. However, it is worth noting that under long-term operating conditions, the microtextures may degrade, which may lead to severe wear and tear. In the existing literature, there are no reports on microtexture degradation in the field of cutting processing, only partial work on the surface texture degradation of tires [196]. Solid lubricants, as the main lubrication method for dry cutting tools, can be used to develop more efficient lubricating materials for tool surfaces. The synergistic effect between biomimetic microtextures and various solid lubricating coatings still needs further exploration. The construction of biomimetic textures with different functions in different areas of the tool is the main research direction for future applications of biomimetic textures on cutting tools.

To date, the true potential of texturing has not been realized for surface texturing, not because of a lack of effective texturing techniques, but because of a serious lack of detailed information on the mechanical function of textures in tribological situations. There is also a lack of important indicators for assessing the performance of surface textures at this stage in terms of the effects they exert [197]. In addition, there is no agreement as yet on the optimum values for the parameters of textures that need to be optimized for surfaces. More importantly, good methods for generating deterministic textures for optimized designs do not exist in practice, and parameter optimization can only be carried out by statistical or mathematical software at this stage. Therefore, significant opportunities remain for advancing microtexture research, including the construction of multi-scale biomimetic microtextures through the synergistic integration of biomimetic shark skin and lotus leaf patterns to enhance debris capture and lubricant retention; leveraging machine learning to predict optimal biomimetic texture parameters in cutting tools for reduced experimental iteration costs; and developing microstructures with precisely engineered geometries to direct cutting fluids toward tool–chip interfaces, thereby minimizing lubricant consumption in aerospace machining processes.

## Figures and Tables

**Table 1:** Advantages and limitations of different texture preparation methods.

Texture Preparation Methods	Advantages	Limitations
Laser ablation [123]	Fast processing speed, flexible operation and good controllability	The heating effect in both methods may lead to problems such as material degradation, and the heat-affected zone will affect the surface topography and mechanical properties of the machined area
Laser interference [87]	Creating textures in high resolution	
Laser impact processing [88]	Produces a surface hardening effect to enhance wear resistance	Need to manufacture microscopic features one by one, the process is less efficient
Reactive ion etching [8,101,102]	Fast etching process and high quality of the prepared textures	This technique results in more visible damage to the material surface, less precise control of the ion beam and harsh experimental environments and expensive equipment
Soft lithography [104,105]	Relatively low cost, easier set-up, higher efficiency and more accurate pattern resolution	Need to use other methods to create stamp masters, such as photolithography or electron beam lithography, and more difficult to create masters for animal body surface patterns
3D printing [114]	Finer parts, patterns and moulds can be constructed and are faster, more flexible and cheaper than traditional techniques	The materials that can be used for 3D printing are very limited, and if the surface of the object to be manufactured is rounded, this can cause deviations in accuracy
